# Fluorescence-Guided Surgery for Hepatoblastoma with Indocyanine Green

**DOI:** 10.3390/cancers11081215

**Published:** 2019-08-20

**Authors:** Yohei Yamada, Michinobu Ohno, Akihiro Fujino, Yutaka Kanamori, Rie Irie, Takako Yoshioka, Osamu Miyazaki, Hajime Uchida, Akinari Fukuda, Seisuke Sakamoto, Mureo Kasahara, Kimikazu Matsumoto, Yasushi Fuchimoto, Ken Hoshino, Tatsuo Kuroda, Tomoro Hishiki

**Affiliations:** 1 Department of Pediatric Surgery, National Center for Child Health and Development, Tokyo 157-0074, Japan; 2 Department of Pediatric Surgery, Keio University School of Medicine, Tokyo 160-8582, Japan; 3 Department of Pathology, National Center for Child Health and Development, Tokyo 157-0074, Japan; 4 Department of Radiology, National Center for Child Health and Development, Tokyo 157-0074, Japan; 5 Center for Organ Transplantation, National Center for Child Health and Development, Tokyo 157-0074, Japan; 6 Children Cancer Center, National Center for Child Health and Development, Tokyo 157-0074, Japan; 7 Department of Pediatric Surgery, International University of Health and Welfare, Chiba 286-0048, Japan; 8 Division of Surgical Oncology, National Center for Child Health and Development, Tokyo 157-0074, Japan

**Keywords:** hepatoblastoma, indocyanine green, near infrared, navigation

## Abstract

Fluorescence-guided surgery with indocyanine green (ICG) for malignant hepatic tumors has been gaining more attention with technical advancements. Since hepatoblastomas (HBs) possess similar features to hepatocellular carcinoma, fluorescence-guided surgery can be used for HBs, as aggressive surgical resection, even for distant metastases of HBs, often contributes positively to R0 (complete) resection and subsequent patient survival. Despite a few caveats, fluorescence-guided surgery allows for the more sensitive identification of lesions that may go undetected by conventional imaging or be invisible macroscopically. This leads to precise resection of distant metastatic tumors as well as primary liver tumors.

## 1. Introduction 

The real-time identification of cancer tissues is desperately needed among surgeons in the field of oncology. First discovered in 1976, protein-bound indocyanine green (ICG) emits light that peaks at approximately 840 nm when illuminated with near-infrared (NIR) light (750–810 nm) [1]. Since then, ICG-guided surgery has been widely applied to fundus angiography in ophthalmology [2], the visualization of the lymphatic flow [3], the identification of sentinel lymph nodes [4], and the assessment of the blood flow in cardiovascular surgery [5] and neurosurgery [6]. 

In the clinical setting, the major advantages of ICG fluorescence imaging are its safety and feasibility. The clinical application of ICG has been approved by the Food and Drug Administration for more than 60 years [7], with a reported incidence of adverse reactions of <0.01% since its approval [8]. 

In 2009, Japanese groups developed intraoperative fluorescence cholangiography by focusing on the biliary excretion of ICG [9]. The advent of real-time cancer visualization in hepatobiliary surgery was actually an incidental product of the intentional application of intraoperative cholangiography, which was originally discovered by Ishizawa et al. [10]. As is well known, ICG is widely used as a reagent to measure the liver function in the field of hepatobiliary surgery. During intraoperative cholangiography, Ishizawa’s group found that the tumors shone bright under NIR light, even before ICG had been injected intraoperatively for cholangiography. It was then discovered that ICG had been administered to these patients two weeks earlier to assess liver function, which led them to hypothesize that ICG was retained longer in cancerous tissues of hepatocellular carcinoma (HCC) than in non-cancerous hepatic parenchyma. Since then, more than 700 adult hepatectomy cases with the utilization of intraoperative fluorescence imaging have been reported. The target diseases have included HCC, colorectal or pancreatic liver metastasis, and intrahepatic cholangiocarcinoma, all of which are well summarized in the review article published in 2018 [11]. 

Hepatoblastomas (HBs), which are the most common pediatric malignant liver tumors and usually diagnosed under three years of age, presumably possess similar features to adult HCC in terms of the ICG uptake and excretion into the biliary system. The histology of HB resembles various stages of the developing liver, including epithelial phenotypes and mesenchymal elements, such as immature fibrous tissue or osteoid [12,13]. Occasionally, an unfavorable subtype of HB presents in older children with clinical and histopathological features reminiscent of HCC [14,15]. Several pilot studies have demonstrated the excellent visualization of HBs under NIR light [16,17]. Furthermore, navigation surgery can be applied to metastatic HB lesions, in contrast to the metastatic lesions of HCCs, since aggressive metastasectomy often provides a survival benefit to HB patients [18,19,20]. 

In this review focusing on ICG fluorescence-guided surgery for HB, the developments, mechanism underlying the ICG uptake, clinical applications, and our experiences are detailed, and future possibilities are discussed.

## 2. The Principles of ICG Imaging for HB

ICG fluorescence imaging of HB has recently been applied for intraoperative navigation. Thus far, no mechanistic analysis has been performed as to why HBs retain ICG longer than normal hepatocytes. However, it can be presumed that HBs possess a similar physiological behavior to HCC concerning the ICG uptake. 

ICG is an organic anion that is almost exclusively taken up by the liver and rapidly excreted into the bile without undergoing biotransformation or enterohepatic circulation [21,22]. ICG is transported by organic anion transporting polypeptide (OATP)-1B3 and Na^+^-taurocholate co-transporting polypeptide (NTCP) [23]. In 2014, Ishizawa et al. conducted a gene set enrichment analysis (GSEA) on HCC samples and identified the association with the ICG fluorescence pattern. Distinct fluorescence patterns of HCC were noted—total type (uniform uptake of ICG) and rim type (ICG uptake only in the superficial area). What they found in the study was that the ratios of NTCP and OATP-1B3 were higher in uniform-type HCC than in rim-type HCC, whereas comparable ratios between these two different fluorescence patterns were noted in the genes associated with excretion. These results led them to speculate that the portal uptake function of ICG is preserved in well-differentiated HCCs, whereas the biliary excretion of ICG is impaired because of morphological changes, leading to the accumulation of ICG in the cancerous tissue [24]. 

Based on these findings, a well-preserved uptake capacity of ICG in HBs and an impaired ability to excrete ICG into the biliary system due to morphological changes leads to a longer retention of ICG in HB tissue than in surrounding non-cancerous tissue, which enables the specific visualization of HBs in NIR mode.

## 3. Modalities

Once tumor tissues take up and retain ICG, exposure to excitation light with 760 nm infrared rays and the collection of emitted fluorescence are all that is needed for visualization. Representative NIR fluorescence devices, which are all commercially available, are described below. The details of each device are beyond the scope of the current review, but each system has its own technical advantages, and equipment-related factors in the setting of intraoperative cholangiography were analyzed recently [25]. While the Photodynamic Eye (PDE) system^®^ is applied in open surgery, all other devices mentioned here are endoscopic types. During open surgery, while surgeons can palpate lesions with the help of fluorescence, surgical light in the operating theater generally must be turned off for the NIR examination. In contrast, in endoscopic surgery, surgeons lose any tactile sensation, but no interference is caused by the room light.

### 3.1. Hamamatsu Photonics: PDE System^®^

A hand-held camera featuring manual adjustment, including an IR excitation wavelength of 760 nm, collects 830 nm wavelength fluorescence emitted by ICG and visualizes it on a monitor in real time. The camera unit of the system encapsulates both an IR radiator and IR camera in the same body. The PDE system is primarily used for open surgery for HCC and HB [10,16]. 

### 3.2. Olympus: VISERA ELITE System^®^

The VISERA ELITE II 3D compatible surgical endoscope system features imaging that can be switched between white and IR light at the push of a conveniently located button. The system enables surgeons to work in two different infrared modes and has been used for lymph node mapping by ICG in laparoscopic surgery for gastric and colorectal cancer [26,27,28]. 

### 3.3. Stryker: PINPOINT System^®^

The PINPOINT system allows for a simultaneous overlay view of the normal white-light mode and NIR mode with the same focal range through a single endoscope, allowing surgeons to perform operations in real time without frequent switching of the screens. This technique has already been applied to laparoscopic cholecystectomy to visualize the biliary anatomy, colorectal surgery to assess the blood supply in the anastomosis, and laparoscopic liver resection to help surgeons identify tumor margin [29,30,31,32]. We reported the first application of this system to metastasectomy in an HB patient [33].

### 3.4. Karl Stolz: D-Light P System^®^

The system includes a light source for visible and 760 nm (ICG mode) light, a plasma light guide, and a 30°/10 mm laparoscope containing optical filters. The system allows for easy switching between white light and ICG modes using a foot pedal. This system has been utilized in laparoscopic hepatectomy for hepatic tumors [34,35] and HB [36].

## 4. Clinical Application of ICG-Guided Surgery for Liver Tumors

### 4.1. Pharmacokinetics of Intravenously Administered ICG

Fluorescence of normal liver tissue develops uniformly over 5–10 minutes if ICG is given intravenously, lasting up to 20–24 h after injection [31,37,38]. In addition, an early study also revealed that hepatocytes in a cirrhotic liver tend to retain ICG longer than normal hepatocytes. In terms of the route of administration, a recent study compared the fluorescence pattern of liver parenchyma following the injection of ICG though the portal vein or a systemic vein. The results revealed essentially no difference in the fluorescence pattern [39]. 

### 4.2. Protocol of ICG-Guided Surgery for HCC and Metastatic Liver Tumors in Adult Patients

In the original ICG-based navigation study for HCC, ICG (0.5 mg/kg) was administered two weeks prior to the operations [10]. In most studies, the interval has ranged from 1 to 14 days [11,40] to obtain the specific visualization of HCC. Since a few instances of no visualization have been reported when ICG was administered too early before the operation [24], such as three weeks or longer, it is safe to say that ICG should be administered within two weeks before surgery. 

Of note, in cases where ICG was given intraoperatively, HCCs and metastatic tumors in the liver appeared as a shadow on the PDE system, i.e., they presented as non-fluorescing spots [26,39]. Ishizawa also mentioned that a two-day interval might be better when applied to patients with advanced cirrhosis, since the signal intensity of the noncancerous liver parenchyma interfered on NIR mode [10]. 

Regarding the optimal dose of ICG, administered doses have ranged from 5 mg/body to 20 mg/body or from 0.25 mg/kg to 0.5 mg/kg [11]. A recent attempt to determine the optimal dose of ICG for delineating liver tumors revealed that much smaller doses (1.25 mg to 2.5 mg/body for adult patients) were sufficient to achieve that goal [41].

In addition, it is important to note that HCCs with different pathologies show different fluorescence patterns. While well- or moderately differenced HCCs tend to show cancerous (diffuse or partial) fluorescence, poorly differentiated HCCs tend to show a rim-type pattern. It is presumed that the expression of transporters associated with the ICG uptake in poorly differentiated HCCs are downregulated in these lesions, as mentioned in a previous study [42]. The fluorescence pattern is also associated with the presence of hepatitis B virus infection, a smaller tumor diameter, and a reduced incidence of microscopic vascular invasion [24]. Given that most HCCs are derived from cirrhotic liver, the interval between the ICG injection and the surgery is recommended to be at least two days, since a higher retention rate of ICG produces a high background intensity, which can interfere with specific visualization.

### 4.3. Institutional Experience

Between 2014 and 2019, at Keio University Hospital and National Center for Child Health and Development, surgeons performed 13 laparotomies for 12 patients with liver resection, as shown in Table 1, 17 thoracotomies for 8 patients with pulmonary metastases, as shown in Table 2, and 6 other surgeries for 5 patients with lymph-node metastasis in the mediastinum (*n* = 1), peritoneal metastases (*n* = 2), pancreatic metastasis (*n* = 1), bone metastasis (*n* = 1), and pleural metastasis (*n* = 1), as shown in Table 3. 

The studies were approved by each institutional review board and informed consents were obtained from all the patients. 

### 4.4. Proposed Protocol of ICG-Guided Surgery for Primary HB Lesions

Only a small series of ICG-guided surgery for primary HB has been reported. In those studies, ICG (0.5 mg/kg) was given 72–96 h prior to the primary hepatectomy in a case reported by Yamamichi et al. [17], 60–138 h early in the cases reported by Souzaki (*n* = 4) [36], and 72 h early in most of our cases (*n* = 12), as shown in Table 4. All of these cases successfully achieved the specific visualization of primary HB lesions as either an uneven or diffuse pattern. Of note, ICG is excreted into the biliary system followed by the bowel loops, so fluorescence in the bowel loops may also produce a non-specific fluorescence. Given that HB occasionally develops lymph node metastasis and dissemination in the abdominal cavity, it would be wise to administer ICG 72–96 h prior to guided surgery in order to minimize the background fluorescence in bowel loops, as by that time, such nonspecific fluorescence will have dissipated. 

**Figure 1:**
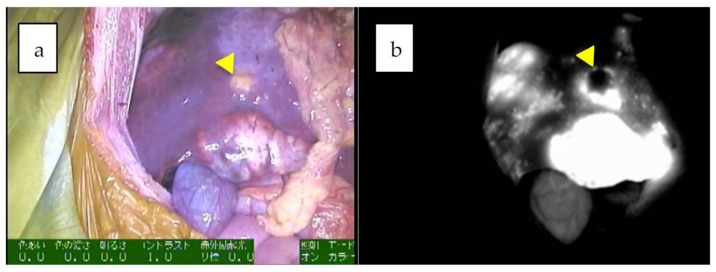
**a**,**b**: Multiple fluorescent spots are observed under near-infrared (NIR) in the liver. Of note, some nodules show a rim-type fluorescence pattern, indicated by an arrowhead (combined fetal and embryonal subtype, post-chemotherapy).

**Figure 2:**
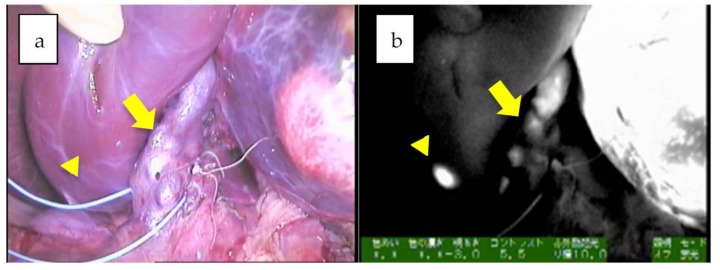
**a**,**b**: The hilum of the liver in Patient 12. A giant tumor with uneven fluorescence in the left lobe along with intrahepatic metastasis (diffuse pattern) is indicated by an arrowhead. Of note, the common bile duct is also visualized, presumably due to residual fluorescence, indicated by an arrow (mixed epithelial and mesenchymal, post-chemotherapy).

**Figure 3:**
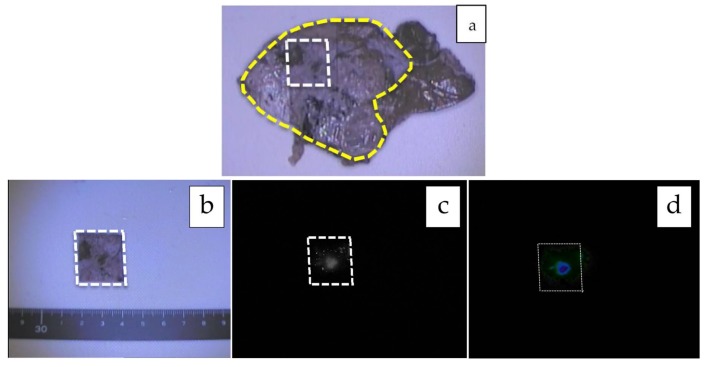

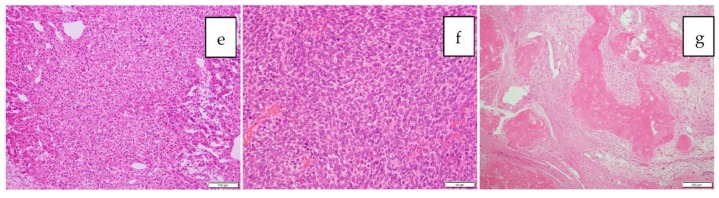
**a**–**g**: Mixed epithelial and mesenchymal type with teratoid features, post-chemotherapy. The formalin-fixed cross section of the liver is shown (**a**), and the tumor is encircled with a yellow-dotted line. The tumor is heterogeneous macroscopically, and fluorescence can be observed only in the small area marked with a white-dotted line (**b**: white-light mode, **c**: NIR mode, **d**: mapping mode). A histological analysis showed that while the fluorescent area corresponded to well-differentiated HB (**e**), the non-fluorescent area consisted of poorly differentiated HB (**f**) and an osteoid lesion (**g**). Scale bar: 100 µm.

**Figure 4:**
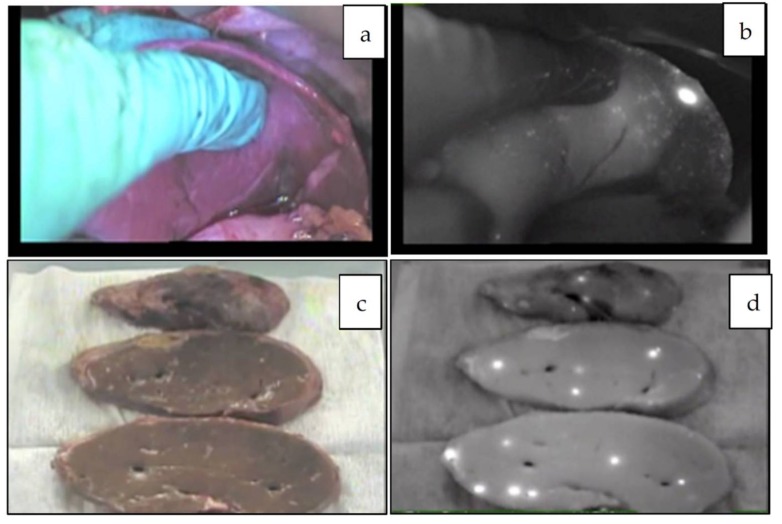
**a**–**d**: Multiple metastatic HBs in the transplanted liver in vivo (**a**,**b**) and ex vivo (**c**,**d**). This patient underwent the second living donor liver transplantation [44].

Since the number of subjects thus far has been small, no clear association has been noted between the fluorescence pattern and pathology. However, it is important to note that all patients with HB in our series underwent sessions of chemotherapy before resection, whereas patients with HCC usually do not. Therefore, all of the primary lesions subjected to the studies were evaluated post-chemotherapy, and a certain percentage of necrotic and fibrotic areas were observed macroscopically. Such areas generally appeared non-fluorescent and were therefore described as an uneven fluorescence pattern in vivo, as shown in Figure 1a,b and Figure 2a,b. Interestingly, HB with teratoid features also demonstrated uneven fluorescence both in vivo and ex vivo, with fluorescence being observed in only well-differentiated HB lesions, as shown in Figure 3a–g. In addition, we experienced cases of metastasectomy for HB in a transplanted liver graft, with multiple small lesions visualized as a diffuse pattern, as shown in Figure 4a–d [43]. 

Based on these findings, the recommended interval between the injection of ICG (0.5 mg/kg) and the operation is approximately 72 h (as some advocate longer intervals may reduce the sensitivity to detect small HBs) when attempting to resect HBs in the liver as fluorescent spots. 

Table 4 shows all of the articles on ICG-fluorescence-guided surgery that have been published in English. 

### 4.5. Proposed Protocol for Metastatic Lesions

#### 4.5.1. Pulmonary Metastases

When resecting pulmonary metastases or other lesions with no interference from residual fluorescence emitted by the liver or bowels, ICG can be given the day before the operation, since there is no concern about background fluorescence. 

**Figure 5:**
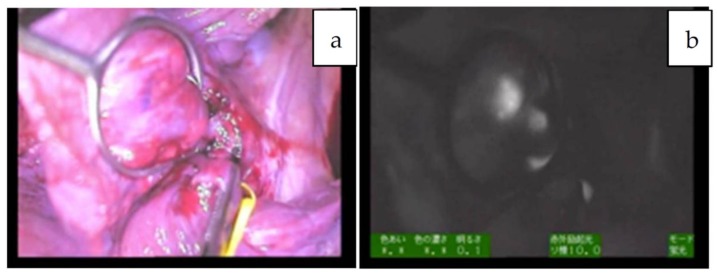
**a**,**b**: Several pulmonary metastases are visualized in NIR mode (patient 19; transitional liver cell tumor).

Representative intraoperative visualizations are shown in Figure 5a,b. In the study conducted by Kitagawa et al., ICG was given 24 h prior to thoracotomy, and they successfully identified 250 fluorescence-positive lesions from 10 different patients across 37 procedures [16]. Among them, the tiniest nodule was as small as 0.062 mm, and the positive predictive value (PPV) was 88.4% [16]. Souzaki et al. performed 6 pulmonary metastasectomies with ICG given 18–27 h prior to the surgeries and found metastases as small as 1.2 mm (100% detectability with 91.6% PPV) [36]. Similarly, Ohno et al. performed eight thoracotomies for six patients, in which ICG was given 24 h before the procedures, and several preoperatively undetected metastatic lesions were newly identified [45]. Of note, there was one false-negative lesion, although why this one metastatic lesion failed to be visualized was unclear, as the same patient underwent another ICG-guided surgery on another occasion, and the metastases were all identified as fluorescent spots using the exact same protocol. The absence of the uptake of ICG in this specific case might be associated with the pathology, as the negative specimen was pathologically different from others resected on different occasions. 

#### 4.5.2. Other Forms of Metastasis

**Figure 6:**
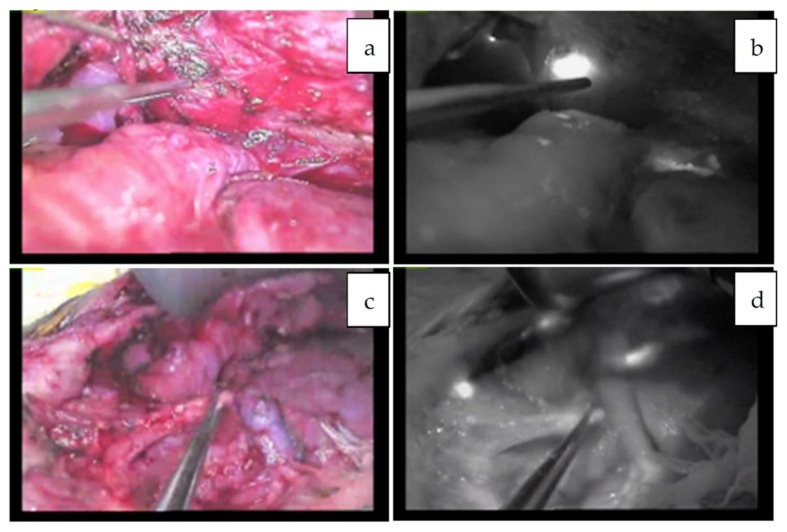
Peritoneal metastases in the patient 8, which were successfully removed with the help of NIR mode. Normal white light mode viewing the abdominal cavity (**a**,**c**) and NIR mode (**b**,**d**).

**Figure 7:**
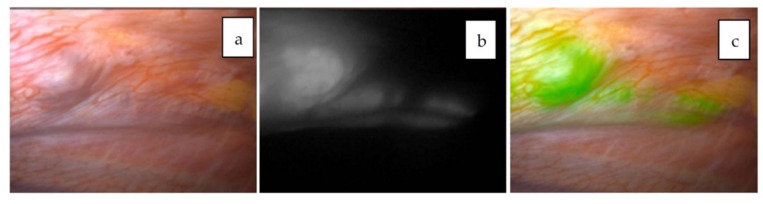
Pleural metastasis visualized with the Pinpoint system. Normal white-light mode (**a**), NIR mode (**b**), and overlay mode (**c**) are shown. The tumor is visualized as a green color overlaid on the white-light mode view.

When metastatic HBs in the abdominal cavity are targeted, such as in cases of disseminated nodules or lymph nodes, the fluorescence excreted into the bowels may interfere with the NIR view. In such situations, ICG should be given around 72–96 h prior to the operation in order to minimize nonspecific fluorescence. For example, ICG was administered 72 h before resection for peritoneal dissemination in patient 8, as shown in Figure 6a–d [44], and for metastases to the pleura and diaphragm in patient 20, as shown in Figure 7a–c [33]. In these cases, fluorescence-guided surgery greatly helped surgeons identify the exact location and extent of the tumor even in a dense, adhesive operative field. The uptake of ICG in metastatic HBs or HCCs is presumed to be mediated by the same transporters as the primary tumors. In contrast, peritoneal metastases from colorectal cancer can be visualized by ICG through the enhanced permeability effect, which is the passage of ICG through abnormal permeable tumoral vessels [46]. 

The duration of ICG retention in metastatic HB is unclear at present. While Satou et al. reported that a disseminated metastatic HCC nodule in the peritoneum fluoresced even 24 days after the injection of ICG [47], the recommended interval is within several days in order to obtain high-contrast fluorescence. 

**Figure 8:**
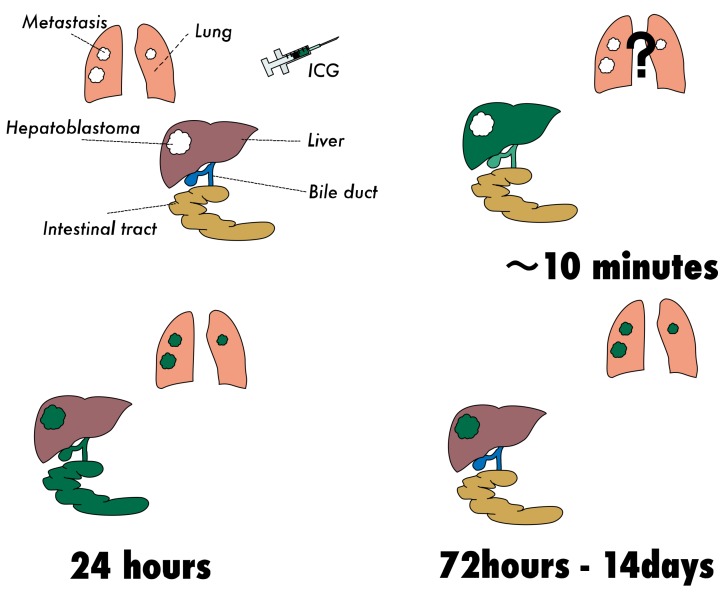
ICG is distributed to the whole body and accumulates in the liver (within a few minutes after the intravenous injection). ICG is then excreted into the biliary system and persists for up to 20–24 h. While liver tumors display non-fluorescent spots in NIR mode at a very early stage after the injection of ICG, such studies have not been performed in lungs with metastatic HBs. The selective retention of ICG can be observed in HBs in both the liver and lungs around 24 h, but excreted fluorescence remains in the bowel loops. By 72–96 h after the injection, bowel-retained ICG is excreted with feces. HB tissues retain ICG for up to two weeks. The pattern of fluorescence may vary depending on the dose of ICG, detecting device, liver function, and pathology.

The schematic description of the ICG uptake by HBs in the liver and lungs is summarized in Figure 8.

## 5. Limitations 

### 5.1. False Positives

Although ICG-guided surgery provides a highly sensitive method for detecting HBs, one potential drawback associated with this approach is the relatively high rate of false positivity. Indeed, several HCC studies utilizing ex vivo specimens revealed a false positive rate of approximately 40% [11]. This high rate of false positivity in these studies may be due to the background cirrhotic liver, in which most cases of HCC develop. No thorough studies regarding false positivity have been performed in primary lesions of HB.

**Figure 9:**
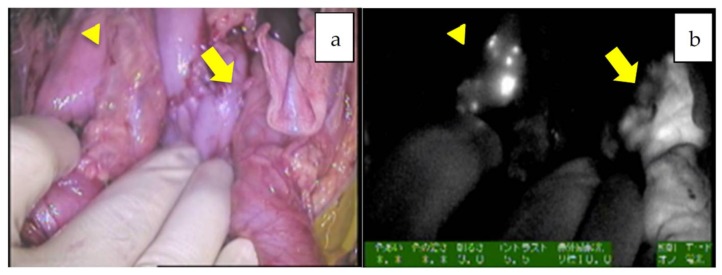
**a**,**b** Non-specific fluorescence in the pancreas and bowel loops. The arrowhead indicates the intense fluorescence in the pancreas, but a thorough inspection denied the presence of metastases. Non-specific fluorescence in the bowl loops was also observed (indicated by an arrow).

The most important point for surgeons is avoiding unnecessary resection in vivo based on such false-positive fluorescence. There are two types of in vivo false positivity. The first one results from surgeons mistaking non-fluorescent spots for fluorescent spots. The frequency of such misperception can be reduced by experience and the presence of a positive control. The vascular green tape made of tetron [48] is useful as a positive control, as it emits light of the same wavelength as ICG. Using this tape, surgeons can compare the visualized targets with a positive control intraoperatively. The second type of false positivity results from the fact that some non-cancerous lesions do indeed take up ICG to some degree. Therefore, surgeons must be aware of the non-specific uptake of ICG in order to avoid causing any serious adverse events through unnecessary resection. Reported ICG-positive noncancerous pathologies in liver include large regenerative nodules, bile duct proliferation, dysplastic nodules, chronic inflammation, fibrosis, normal liver parenchyma, bile plug, and cysts [10,24,42,49]. We have also experienced the non-specific uptake of fluorescence in the pancreas as well, as shown in Figure 9a,b, possibly due to residual ICG in the intrapancreatic bile duct. It would be prudent to consider additional resection only if newly detected fluorescent lesions in the liver or other organs are confirmed by intraoperative ultrasonography or a thorough inspection and palpation. 

Reported non-cancerous pathologies in the lungs include alveolar cells, thrombosis, necrotic tissues, and granulomas [16,45] At present, it is preferable to palpate any suspicious fluorescent lesions in the lungs before performing resection and only resect them if it is technically safe and feasible, according to the surgeon’s discretion.

Several attempts have been made to quantify the intensity of fluorescence and determine the cut-off value for differentiating cancerous from noncancerous tissues. Ishizawa et al. [24] compared the fluorescence intensities of grossly unidentifiable HCCs with non-cancerous lesions using a software program (U11437; Hamamatsu Photonics, Hamamatsu, Japan), which revealed significantly higher fluorescence intensities in HCCs than non-cancerous lesions. Morita et al. also quantified the intraoperatively newly detected lesions’ fluorescence using the Scion image software program (Scion Corporation, Frederick, MD, USA) [42]. Their analyses revealed that false-positive lesions tend to have higher-intensity fluorescence than HCCs. While these findings may seem contradictory, this may be due to the fact that most of the HCCs assessed in the study displayed an uneven emission pattern, whereas most benign lesions displayed a homogeneous intra-lesion emission pattern. It is important to note that these analyses were only performed in ex vivo specimens, not in vivo targets. In vivo quantification is technically more complicated and impractical with currently available devices than an ex vivo approach.

### 5.2. Depth

Another technical limitation lies in the fact that the fluorescence emitted from ICG penetrates only 5–10 mm into tissue. Therefore, any lesions more deeply located are missed by current devices. To address this issue, photoacoustic imaging technology is being developed, which may enable the simultaneous visualization of the ICG accumulation using images obtained from intraoperative ultrasonography [50]. In their study, Miyata et al. investigated the use of a novel type of photoacoustic tomography using ICG as a contrast with the aim to visualize deeply located tumors measuring the ICG uptake. While further technical improvement is needed, photoacoustic tomography is a promising technique that may prove useful in the future.

## 6. Conclusions and Future Prospects

Whether it be in open or endoscopic surgery, real-time cancer navigation for HBs is sure to become a routine procedure, as it is a powerful tool for identifying the location and extent of HBs with just a single injection of ICG. However, surgeons must be aware of the limitations mentioned in this review. Newer optical techniques with automated standardization and allowing for deeper exploration are warranted.

Importantly, whether or not ICG-guided surgeries actually improve the postoperative prognosis remains to be clarified. While the early recurrence rate tended to be reduced after the introduction of ICG fluorography in a study of HCC, no survival benefits have yet been observed [42]. Since the incidence of HB is much lower than that of HCC, a retrospective prognostic analysis with propensity score matching will be the first attempt to clarify this point. 

## Figures and Tables

**Table 1:** Thirteen liver resections for 11 patients.

Case No	Patient	Age/Sex	Device	Procedure	Primary Pathology	Fluorescence Pattern	Tumor Size	Preoperative AFP ^3^ (ng/dL)	Usefulness and Disease Free Survival (Months)
1	1	1y/F	PDE ^1^ neo system	left lobectomy	mixed epithelial and mesenchymal type with teratoid features, post-chemotherapy	uneven	125 × 75 × 70 mm	87.8	identification, surgical margin, 20 m
2	2	2y/M	PDE neo system	LDLT ^2^	combined fetal and embryonal subtype, post-chemotherapy	uneven	40 mm, 70 mm	5514.4	identification, surgical margin, 29 m
3	3	8m/M	PDE neo system	right lobectomy	fetal, post-chemotherapy	uneven	65 mm	41,311	identification, surgical margin, 18 m
4	4	1y/F	PDE neo system	right trisegmentectomy	combined fetal and embryonal subtype, post-chemotherapy	uneven	65 mm	936.3	identification, surgical margin, 17 m
5	5	4y/M	PDE neo system	LDLT ^2^	combined fetal and embryonal subtype, post-chemotherapy	uneven	90 mm	42591	identification, surgical margin, newly detected tumors, 19 m
6	6	2y/M	PDE neo system	LDLT ^2^	embryonal, post-chemotherapy	uneven	25 mm, 10 mm	6.6	identification, surgical margin, not disease free
7	7	9m/M	PDE neo system	left lobectomy	mixed fetal and embryonal subtype, post-chemotherapy	uneven	51 × 66 × 52 mm	46.1	identification, surgical margin, 3 m
8	8	14y/M	PDE neo system	partial resection (recurrences in the transplanted liver)	wholly epithelial type and fetal subtype	diffuse	8 mm	69	identification, surgical margin, newly detected tumors
9	8	14y/M	PDE neo system	LDLT ^2^	wholly epithelial type and fetal subtype	diffuse	8 mm	394	identification, surgical margin, 34 m
10	9	8m/F	PDE neo system	right lobectomy	mixed epithelial and mesenchymal, post-chemotherapy	uneven	22 × 17 mm	1340	identification, surgical margin, 26 m
11	10	1y/M	PDE neo system	right lobectomy	fetal, post-chemotherapy	uneven	128 × 88 × 70 mm	807	identification, surgical margin, 7 m
12	11	1y/M	PDE neo system	left lateral segmentectomy	fetal	uneven	32 × 25 × 20 mm	361.5	identification, surgical margin, 1 m
13	12	4y/M	PDE neo system	LDLT ^2^	mixed epithelial and mesenchymal, post-chemotherapy	uneven	130 × 115 × 90 mm	545323	identification, surgical margin, false positive, 8 m

^1^ PDE: Photo Dynamic Eye, ^2^ LDLT: living donor liver transplantation, ^3^ AFP: Alphafetoprotein.

**Table 2:** Seventeen thoracotomies for eight patients with pulmonary metastases.

Case No	Patient	Age/Sex	Device	Procedure	Primary Pathology	Fluorescence Pattern	Tumor Size	Preoperative AFP (ng/dL)	Usefulness and Disease Free Survival (Months)
1	13	3y/M	PDE neo system	right wedge resection	NA ^1^	diffuse	6 mm	168.8	identification, false positive
2	13	3y/M	PDE neo system	left wedge resection	NA ^1^	diffuse	7 mm	653.1	identification
3	13	4y/M	PDE neo system	right wedge resection	NA ^1^	diffuse	7 mm	101.7	identification, 44 m
4	14	6m/M	PDE neo, D-light	right and left wedge resection	mixed epithelial and mesenchymal type, simple subtype	diffuse	left 1.5 mm, right 1 mm	11623.9	identification, newly detected tumors, false positive
5	14	10 m/M	PDE neo system	right and left wedge resection	mixed epithelial and mesenchymal type, simple subtype	diffuse	6.7 mm	963.1	identification, newly detected tumors, false positive, 34 m
6	15	7y/M	PDE neo system	right wedge resection	mixed fetal + embryonal	diffuse	3.5 mm, 2.8 mm, 1 mm, 3 mm, 3 mm	3428.3	identification, newly detected tumors, false positive, 34 m
7	16	11y/M	PDE neo system	right wedge resection	mixed epithelial and mesenchymal type	diffuse	5 mm	146324	identification, false positive
8	16	11y/M	PDE neo system	right and left wedge resection	mixed epithelial and mesenchymal type	diffuse	left 4 mm, right 1mm	123.1	identification
9	16	13y/M	PDE neo system	right wedge resection	mixed epithelial and mesenchymal type	diffuse	5 mm	354.2	identification, newly detected tumors, 11 m
10	17	12y/f	PDE neo system	left wedge resection	NA ^1^	diffuse	13 mm	16.2	identification, 28 m
11	18	3y/M	PDE neo system	right wedge resection	embryonal	diffuse	2 mm	1154	identification, newly detected tumors, false positive, 45 m
12	2	2y/M	PDE neo system	right and left wedge resection	combined fetal and embryonal subtype	diffuse	left 8, 6, 3, 7, 4 mm, right 2, 3, 1 mm	11492.6	identification, newly detected tumors
13	2	4y/M	PDE neo system	right wedge resection	combined fetal and embryonal subtype	false negative	2 mm	31.5	false negative
14	2	4y/M	PDE neo system	right and left wedge resection	combined fetal and embryonal subtype	diffuse	left 3 mm, right 2–3 mm	66.1	identification, newly detected tumors
15	2	5y/M	PDE neo system	right wedge resection	combined fetal and embryonal subtype	diffuse	10 mm, 3.5 mm	95.8	identification, newly detected tumors, 1m
16	19	20y/F	PDE neo system	right wedge resections	transitional liver cell tumor	diffuse	9 mm	3525	identification, newly detected tumors
17	19	21y/F	PDE neo system	right upper +middle lobectomy	transitional liver cell tumor	diffuse	12 mm	411	identification, 23 m

^1^ NA: not available.

**Table 3:** Other surgeries for distant metastases, except for those to the lungs. HB: hepatoblastoma.

Case No	Patient	Age/Sex	Device	Procedure	Primary Pathology	Fluorescence Pattern	Tumor Size	Preoperative AFP (ng/dL)	Usefulness and Disease Free Survival (Months)
1	16	13y/M	PDE neo system	Lymphadenectomy at tracheal bifurcation	mixed epithelial and mesenchymal type	diffuse	18 × 10 × 31 mm	354.2	identification, 11 m
2	1	2y/F	PDE neo system	Distal pancreatectomy + lymphadenectomy	HB with teratoid features	negative	16 × 16 mm	5.8	NA, 6 m
3	1	1y/F	PDE neo system	Bone biopsy	HB with teratoid features	negative	NA	643.7	identification
4	8	14y/M	PDE neo system	Resection for peritoneal nodules	wholly epithelial type and fetal subtype	diffuse	undetected	69	identification, 34 m
5	15	7y/M	PDE neo system	Resection for peritoneal nodules	NA	diffuse	21 × 14 × 18 mm	525.6	identification, tumor margin, not disease free
6	20	19y/F	Pinpoint + PDE neo	Pleural and diaphragm resection	wholly epithelial type and fetal subtype	diffuse	47 × 38 × 21 mm	2885	tumor margin, identification, 28 m

**Table 4:** Previous reports on indocyanine green (ICG)-fluorescence-guided navigation for HB. PPV: positive predictive value.

References	Number of Patients	Location	Surgical Approach/Device	ICG administration Route/Dose/Timing Prior to Operation	Cancer Detectability
Kitagawa N, 2015 [16]	10	Lungs	Open/PDE (Hamamatsu Photonics)	IV, 0.5 mg/kg, 24 h	250 pulmonary metastases were identified and extirpated. PPV = 88.4%
Yamamichi T, 2015 [17]	3	Primary	Open/HyperEye Medical System MNIRC-1000 (MIZUHO Medical Co. Ltd)	IV, 0.5 mg/kg, 72–96 h	Tumor identification, tumor margin
Toyofumi F, 2017 [43]	1	Lungs	Open/Medical Imaging Projection System, (Panasonic AVC Networks)	IV, 0.5 mg/kg, 24 h	Tumor identification
Yamada Y, 2018 [33]	1	Pleura and diaphragm	Endoscope/Pinpoint System (Stryker)	IV, 0.5 mg/kg, 72 h	Tumor identification, tumor margin
Souzaki R, 2019 [36]	5	Primary and lung	Endoscope/D-Light P (Karl Storz)	IV, 0.5 mg/kg, 18-27 h (lungs), 60–138 h (primary)	Tumor identification, tumor margin, PPV = 91.6%
Takahashi N, 2019 [44]	1	Peritoneum	Open/PDE (Hamamatsu Photonics)	IV, 0.5 mg/kg, 72 h	Tumor identification, tumor margin
